# Screening and identification of versican as a sensitive biomarker and potential therapeutic target in basal cell carcinoma

**DOI:** 10.7150/ijms.105650

**Published:** 2025-04-28

**Authors:** Wenlin Li, Yang Wang, Qiang Hu, Sainan Li, Dachuan Guo, Lin Liu, Xiuqing Huang, Lin Dou, Qi Zhou, Tao Shen, Jianmin Chang

**Affiliations:** 1 Department of Dermatology, Beijing Hospital, National Center of Gerontology, Institute of Geriatric Medicine, Chinese Academy of Medical Sciences, Beijing, China.; 2 The Key Laboratory of Geriatrics, Beijing Institute of Geriatrics, Institute of Geriatric Medicine, Chinese Academy of Medical Sciences, Beijing Hospital/National Center of Gerontology of National Health Commission, Beijing 100730, China.; 3 School of life sciences, North China University of Science and Technology, Tangshan 063210, China.; 4 Peking Union Medical College, Chinese Academy of Medical Sciences, Graduate School of Peking Union Medical College, Beijing, China.

**Keywords:** Basal cell carcinoma, Tissue-specific key genes, Bioinformatics analysis, VCAN, differentially expressed genes

## Abstract

Basal cell carcinoma (BCC) is the most common type of non-melanoma skin cancer (NMSC). However, few biomarkers have been developed for the diagnosis of BCC. This study aimed to uncover novel BCC biomarkers for diagnosis and treatment via transcriptome and pathogenesis investigations. Microarray datasets of BCC tissues were downloaded from the Gene Expression Omnibus (GEO) database and differentially expressed genes (DEGs) were identified. A total of 558 DEGs were identified between BCC and normal samples from the GSE125285 and GSE42109 datasets. 69 DEGs were expressed in a tissue/organ-specific manner, of which three tissue-specific key genes were finally identified. The three genes showed high performance for BCC diagnosis with AUCs of ≥0.8, indicating that they have high diagnostic significance. CIBERSORT analysis revealed an increase in resting NK cells, M1 macrophages and a decrease in dendritic cells in the immune microenvironment of BCC patients. In addition, versican (VCAN) may be involved in the polarization of M1 macrophages in skin cancer. When VCAN was knocked down, the skin cancer cell line A431 was weakened in terms of proliferation, migration and invasion. Meanwhile, the expression of the key oncogenic factors DDX5 was also reduced and apoptosis was promoted through the BAX/BCL-2/c-Caspase3 pathway. The cell-derived xenograft model study in nude mice showed that knockdown of VCAN in tumour cells significantly suppressed tumour size compared to control tumour cells, suggesting that VCAN is one of the important genes for tumourigenesis. Meanwhile, we examined the level of macrophage M1 polarization in tumour samples from a cell-derived xenograft mouse model, and VCAN knockdown significantly reduced macrophage M1 polarization compared to controls. We also detected the expression of VCAN in tumour samples from BCC patients and verified that VCAN expression was significantly higher in BCC than in normal skin tissue. Thus, VCAN could be a potential clinical target for the diagnosis and treatment of BCC.

## Background

BCC is the most common type of skin malignancy, accounting for approximately 85% of non-melanoma skin cancer (NMSC). BCC is more prevalent in older people and has a higher incidence and mortality rate among Caucasians, occurring in the head, face, neck and back of hands, especially in the prominent areas of the face. Risk factors for BCC have been identified, including ultraviolet exposure, ionizing radiation, immunosuppressant, and advanced age 1, 2. Once BCC metastasizes, the effectiveness of surgical treatment is significantly reduced, and patients have a poor prognosis. Due to the lack of specific biomarkers, BCC often remains undiagnosed in a timely manner, leading to delays in relevant treatments 3. Therefore, the identification of specific biomarkers is crucial for the early diagnosis and treatment of BCC.

Recent studies have shown that many proteoglycans are involved in tumour development and progression. Versican (VCAN), an extracellular matrix proteoglycan, plays a pivotal role in several aspects of cancer development, such as proliferative signalling, evasion of anti-growth signaling, tissue invasion and metastasis, regulation of cell death, and promotion of neoangiogenesis 4, 5. VCAN is highly expressed in most tumour tissues and tumour-associated stromal tissues 4, 6, 7. The core protein of VCAN consists of an N-terminal G1 structural domain, a C-terminal G3 structural domain, and a chondroitin sulfate chain-binding region. Five splice isoforms of VCAN have been identified 4, 6. The human immune system plays a crucial role in combating cancer. Immune checkpoint genes such as PD-1, PDL-1, and CTLA-4 are of vital importance in maintaining self-immune tolerance and preventing the occurrence of autoimmunity, protecting organisms from immune responses against themselves. However, cancer cells can take advantage of the characteristics of PD-1 to avoid being detected and attacked by the immune system. PD-1 inhibitors have a significant impact on tumour immunotherapy by enhancing T-cell responses 8-10. Current research has found that VCAN is closely related to the expression of immune checkpoint genes and tumour mutation burden, and it may be a biomarker for the sensitivity of immune checkpoint (PD-1/CTLA-4) inhibitors 11. This indicates that VCAN may have a biological role in tumour immunology, especially in the signaling pathways related to immune checkpoints.

Based on the screening criteria, DEGs were identified and tissue/organ specific expressed genes were discovered. GO and KEGG analyses were then performed and a protein-protein interaction (PPI) network was constructed. Meanwhile, the distribution characteristics of 22 immune cells were analysed, and Pearson correlation coefficients of 22 immune cells were calculated to analyse the co-expression patterns of infiltrating immune cells. We then validated the screened hub genes using different GEO datasets. Finally, knockdown of the hub gene VCAN detects the progression of BCC *in vitro* and *in vivo* covering proliferation, invasion and migration. Cell-derived xenograft mouse model and BCC patient tumour samples were used to verify the results of transcriptomic and cytological experiments. This study reveals the pathogenesis and pathological process of BCC at the transcriptional level and explores potential biomarkers for the diagnosis and differential diagnosis of BCC.

## Material and methods

### Microarray data acquisition and identification of DEGs

Gene expression data were obtained from the GEO database (http://www.ncbi.nlm.nih.gov/geo) 12. Screening criteria included: (1) Homo sapiens expression profiling by array; (2) datasets with more than ten samples; (3) tissue from BCC or normal skin from biopsies; (4) one biopsy sample per subject was analysed without replication 13. The miRNA datasets GSE34535 and GSE34137 were analyzed, where GSE34535 included seven control samples and seven BCC samples, and GSE34137 contains six BCC samples and six control samples. The mRNA datasets are GSE125285 and GSE42109, where GSE125285 includes 25 control samples and 25 BCC samples, and GSE42109 contains 11 BCC samples and 10 control samples. The validation mRNA dataset GSE53462 contains 5 control samples and 16 BCC samples **(Table 1)**.

In order to better visualize these DEGs, gene analysis of inter-sample identification was performed using the 'limma' package in the R language (v.4.2.2) 14. For data visualisation, heat maps and volcano plots were generated using the 'ggplot2' package in the R language. Screening criteria were log2 (fold change) > 1 or < -1 and *P* value < 0.05 15, 16.

### Identification of the tissue/organ-specific expressed genes

Tissue distribution was analysed using the online tool BioGPS (http://biogps.org/) to better understand the tissue/organ specific expression of these DEGs 17. Screening criteria included: (1) the average level of gene expression was 10 times higher in a single tissue or organ; (2) the second highest level of expression was less than one third of the highest level 13.

### Enrichment analysis and construction of the PPI network

The DAVID website (https://david.ncifcrf.gov/) was used for functional enrichment analysis and gene annotation of DEGs 18. The GO annotation analysis included biological processes (BP), cellular components (CC) and molecular functions (MF), and the KEGG pathway analysis included signalling pathways. Enrichment function between DEGs was determined by GO and KEGG enrichment analysis, and *P* < 0.05 was used as the threshold for GO and KEGG enrichment analysis. Gene Set Enrichment Analysis (GSEA, v.4.3.2) was used to assess the distribution trend of genes of a predefined set in the gene table to determine their contribution to the phenotype 19.

Based on all DEGs, the PPI network was constructed using the online tool String (https://cn.string-db.org/) with adjusted *P* < 0.05 20. The interaction information was then downloaded and the PPI network was visualised using Cytoscape software (v.3.8.0) 21. Molecular Complex Detection (MCODE) was used to identify significant gene clusters and obtain cluster scores (filter criteria: degree cut-off = 2; node score cut-off = 0.2; k-core = 2; max depth = 100). CytoHubba was used to identify significant genes as hub genes 22. Five algorithms, namely Degree, Maximum Clique Centrality (MCC), Maximum Neighbourhood Component (MNC), Density of Maximum Neighbourhood Component (DMNC) and Edge Percolated Component (EPC), were used to calculate the top 40 hub genes 13. Finally, all results were intersected to obtain the final hub genes.

We performed enrichment analysis using the Cluego plugin as a way to verify the biological function of the key tissue-specific genes in BCC 23. Only pathways with *P* < 0.05 were be included and using GO term fusion. GO tree intervals were selected from 3 to 8 and specificity was set to a medium level.

### Tumour-infiltrating immune cell analysis

Based on the GSE125285 dataset, the CIBERSORTx website (https://cibersortx.stanford.edu/) was used to calculate the abundance of 22 types of immune cell types in BCC and normal tissue 24. The inverse fold-processing method of the CIBERSORTx website was used to calculate the distribution profiles of 22 immune cell types, including activated dendritic cells (DCs), resting DCs, activated mast cells, resting mast cells, activated natural killer cells (NKs), resting NKs, activated memory CD4+ T cells, resting CD4+ T cells, naive CD4+ T cells, regulatory T cells (Tregs), T follicular helper cells (Tfhs), gamma delta T cells (Tgds), CD8+ T cells, eosinophils, neutrophils, monocytes, macrophages (M0s), type 1 macrophages (M1), type 2 macrophages (M2), memory B cells, naive B cells, and plasma cells. Histograms and heat maps show the infiltration rates of immune cells in different samples. Co-expression patterns of immune-associated DEGs and infiltrating immune cells were analysed using Pearson correlation coefficients with the “corrplot” package. Immune-related genes were obtained from the Immunology Database and Analysis Portal (IMMPORT, http://www.immport.org/) 25.

### Construction of ceRNA network

TargetScan (https://www.targetscan.org/) 26, miRTarBase (https://mirtarbase.cuhk.edu.cn/) 27, miRBase (http://www.mirbase.org/) 28, and miRDB (http://www.mirdb.org/) 29 were used to predict the interactions of mRNA-miRNA. The interaction networks were visualized using Cytoscape software for the ceRNA network.

### Cell cultures and treatments

A431 cells were cultured in Dulbecco's Modified Eagle Medium (DMEM) containing 10% fetal bovine serum (FBS) and antibiotics (100 U/mL penicillin G and 0.1 mg/mL streptomycin) at 37 °C in an incubator with 5% CO_2_ and 95% air (v/v). At the start of each experiment, the plated cells were first cultured in DMEM medium with 10% FBS for 24 h, then synchronized with DMEM medium containing 0.5% FBS for 24 h, and subsequently treated with doxorubicin (DOX) for 24 h.

Human siRNA Negative control (Nci, Cat. No. sc-37007, Santa Cruz, USA) and human VCAN siRNA (Cat. No. sc-41903, Santa Cruz, USA) were purchased from Santa Cruz Co. When the cell density was cultured to 70%, Nci and si-VCAN were transfected into cells using HiPerFect transfection reagent (QIAGEN). After 48 hours of transfection, the transfected cells were replaced with fresh DMEM medium. The cells were then pretreated with DOX for 24 hours.

Cell-Counting-Kit-8 (CCK8, Solarbio, China) was used to measure the number of live cells as previously described. Briefly, 10 μL of CCK8 solution was added to a 96-well plate seeded with cells and incubated for 4 hours. The absorbance was recorded at 450 nm.

### Cell derived xenograft

Control lentiviral particles (sc-437282) and VCAN shRNA lentiviral particles (sc-41903-V) were purchased from Santa Cruz Biotechnology. A431 cells were infected with control or VCAN shRNA lentivirus, the VCAN shRNA lentivirus infected cells were selected with 5 μg/mL puromycin for 2 weeks to obtain a stable VCAN knockdown cell line. Male Balb/c nude mice aged 8-10 weeks were purchased from SPF (Beijing) Biotechnology Co. Nude mice were injected subcutaneously in the axillary region with 5 × 10^6^ control cells (sh-NC) or a VCAN knockout cells (sh-VCAN). On day 14, nude mice were euthanized and body and tumour weights were determined.

### Wound healing assay

Cells were seeded in a 6-well plate at a density of 5 × 10^5^ per well and grown to 80-100 % confluence. A straight scratch was made with a pipette tip to stimulate a wound. The cells were then washed 3 times with phosphate buffered saline (PBS) to remove the debris and replaced with serum-free medium for 24 h at 37 °C. The width of the scratch gap was recorded using an inverted microscope and underwent the photographing process at 0, 24, 48 and 72 h. Each experimental procedure was performed in triplicate for differences between the 0 h wound width and the width of the quantitative cell migration process.

### Transwell migration and invasion assay

After trypsinisation and rinsing with PBS, cells (2 × 10^5^/mL) were suspended in serum-free DMEM medium. The cells were then seeded into the upper filter of a 12-well transwell chamber, which had previously been coated with diluted Matrigel. The transwell chamber was seeded with Matrigel mix (BD Biosciences, San Jose, CA, USA) for invasion assays and without Matrigel mix for migration assays. The 10% FBS medium added to the lower chamber medium was used as a source of chemoattractants. Cells were cultured at 37°C for 24 h. Cells passing through the membrane were fixed with 4% paraformaldehyde for 20 min and stained with 0.1% crystal violet for 30 min at room temperature. The cells were observed under an inverted microscope 30.

### Western blotting assay

A431 cells were lysed with lysis buffer containing a complete protease inhibitor and phosphatase inhibitor. Protein concentration was determined by the BCA method. Samples were loaded on SDS-PAGE for electrophoresis and transferred to PVDF membranes. Each PVDF membrane was cut horizontally to analyse 2-4 proteins of different molecular weight, blocked with 5% skim milk, incubated with primary antibody overnight at 4℃, washed with TBST, incubated with secondary antibody and detected with enhanced chemiluminescence reagent. If there are multiple proteins in an image that need to be detected simultaneously, we use 2-3 PVDF membranes for separate detection and detect GAPDH on each membrane simultaneously. When displaying the images, we selected one of the PVDF membranes for GAPDH from the same batch of samples as a representative display. GAPDH was used as a protein loading control. Finally, densitometric analysis was performed using ImageJ software (http://rsb.info.nih.gov/ij/) as previously reported 31.

### Immunohistochemistry (IHC) and immunofluorescence (IF)

Tissue samples were fixed in 4% paraformaldehyde, dehydrated and embedded in paraffin. 5 μm sections were cut and mounted on poly-L-lysine slides.

Immunohistochemistry: Sections were deparaffinised in xylene, rehydrated and antigen retrieved with citrate buffer (pH 6.0) by microwave for 10 min. Endogenous peroxidase was quenched with 3% hydrogen peroxide for 15 min. After PBS washes, primary antibodies were incubated overnight at 4°C. After PBS washing, biotinylated secondary antibodies were added for 1 h at room temperature. Immunoreactivity was visualised using a DAB kit and sections were counterstained with haematoxylin, dehydrated, cleared and mounted. Sections were examined under an inverted light microscope. VCAN polyclonal antibody was purchased from Proteintech Group, Inc (Cat No.30599-1-AP).

Immunofluorescence: After antigen retrieval, sections were blocked with 10% normal goat serum for 1 hour at room temperature. Primary antibodies were incubated overnight at 4°C. After PBS washes, fluorescent secondary antibodies were incubated for 1 h in the dark. Cell nuclei were counterstained with DAPI. Sections were mounted with antifade medium and examined by fluorescence microscopy. Anti-iNOS Mouse mAb was purchased from Servicebio (Cat No. GB123965-50). 31

### Statistics analysis

IBM SPSS Statistics 25.0 was used for statistical analysis and ROC curve plotting. Student's t-test was used to compare differences between the two groups. Circle graph and Violin Plot were plotted using https://www.bioinformatics.com.cn, an online platform for data analysis and visualisation. GraphPad Prism 8.0.2 was used for statistical analysis and image construction. Adobe Illustrator 2022 software was used for figure editing 25.

## Results

### Identification of DEGs

Compared to genes in normal skin samples, a total of 2450 de-mRNAs were found in the GSE125285 dataset and a total of 1982 de-mRNAs were found in the GSE42109 dataset. There were 558 differentially expressed genes common to both datasets. We identified a total of 85 de-miRNAs in datasets GSE34535 and a total of 89 de-miRNAs in datasets GSE34137. There were 25 co-differentially expressed de-miRNAs between the two datasets.

### Identification of the tissue/organ-specific expressed genes

A total of 69 tissue/organ-specific expressed genes were identified by BioGPS. Most of these genes were expressed specifically in the haematological/immune system (n = 15, 21.74%). The second and third organ-specific expression systems were the nervous system (n = 14, 20.29%) and the respiratory system (9/69, 13.04%). This was followed by placenta (n = 7, 10.14%), the endocrine system (n = 6, 8.70%), muscle (n = 6, 8.70%), the digestive system (n = 5, 7.25%) (Table 2).

### Enrichment analysis and construction of the PPI network

**Figure 1:**
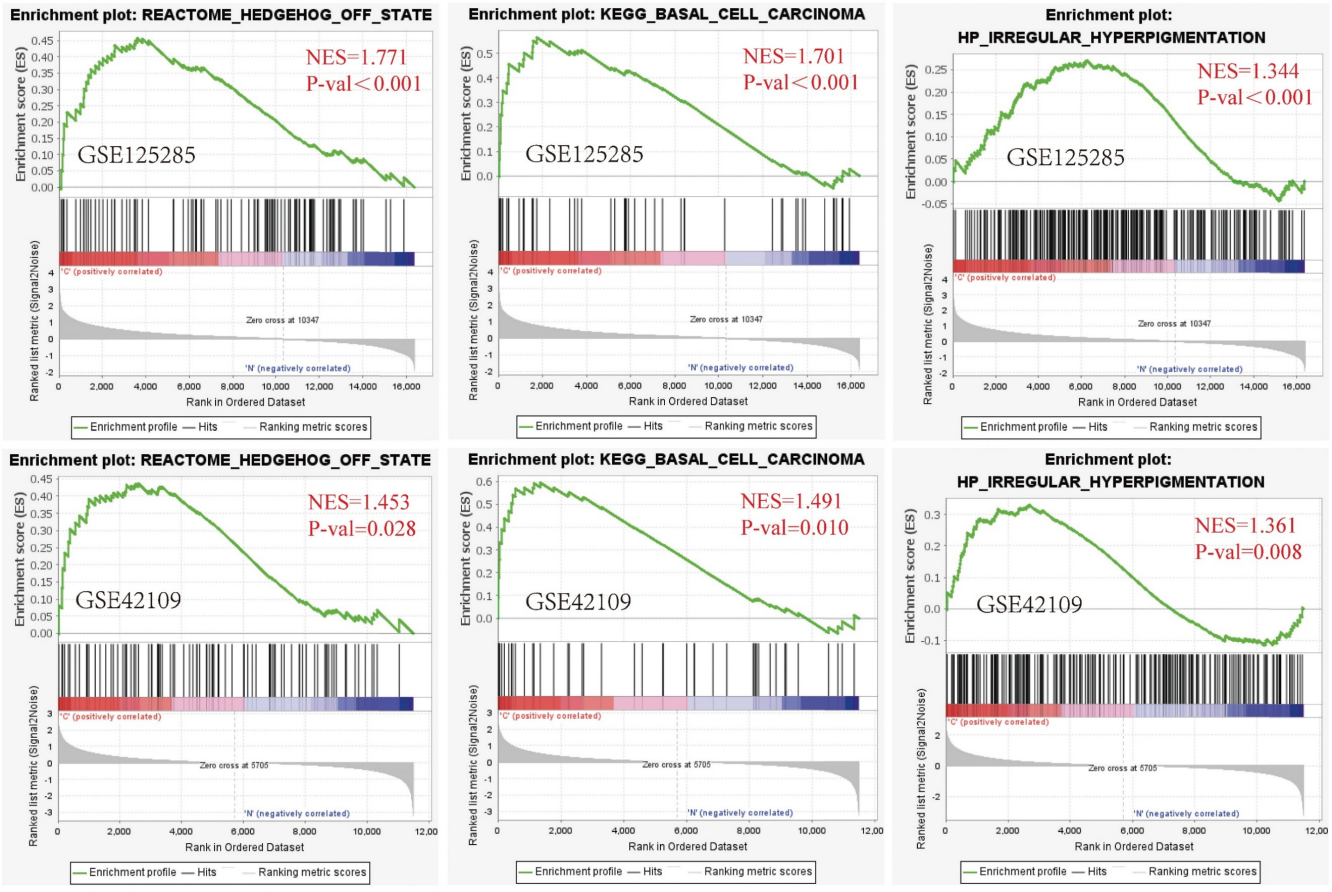
GSEA plot showing the most enriched BCC-related genes sets in the dataset GSE125285 and GSE42109. From left to right: Gene set enrichment analysis: REACTOME HEDGEHOG OFF STATE; Gene set enrichment analysis: KEGG BASAL CELL CARCINOMA; Gene set enrichment analysis: HP IRREGULAR HYPERPIGMENTATION (above: GSE125285, below: GSE42109). The screening criteria for significant gene sets were *P* < 0.05 and Q < 0.25. NES: normalized enrichment score; *P*-val: False Discovery Rates (FDR)or adjust* P* value.

GSEA software was utilized for gene set enrichment analysis of the GSE125285 dataset, which was secondarily validated by the GSE42109 dataset (NES > 1, *P* value < 0.05). After analysis of the GSE42109 dataset, the four most highly correlated enrichment pathways were PID HEDGEHOG 2PATHWAY (FDR = 0.197, *P* < 0. 001), REACTOME HEDGEHOG OFF STATE (FDR=0.099, *P*<0.001), REACTOME HEDGEHOG ON STATE (FDR=0.066, *P*<0.001) and KEGG HEDGEHOG SIGNALING PATHWAY (FDR = 0.049, *P* < 0.001). These pathways were all associated with the HEDGEHOG signalling pathway, indicating that the HEDGEHOG signalling pathway plays a very important role in BCC development. Second, two gene sets associated with BCC were also significantly altered, namely KEGG BASAL CELL CARCINOMA (FDR = 0.060, *P* < 0.001), HP BASAL CELL CARCINOMA (FDR = 0.050, *P* < 0.001). There were gene sets enriched for altered skin properties such as HP IRREGULAR HYPERPIGMENTATION (FDR=0.051, *P*<0.001) associated with excessive irregular pigmentation, MURAKAMI UV RESPONSE 24HR (FDR=0.060, *P*<0.001) associated with the relationship between UV and skin keratin formation (Fig. 1, Fig. S2).

**Figure 2:**
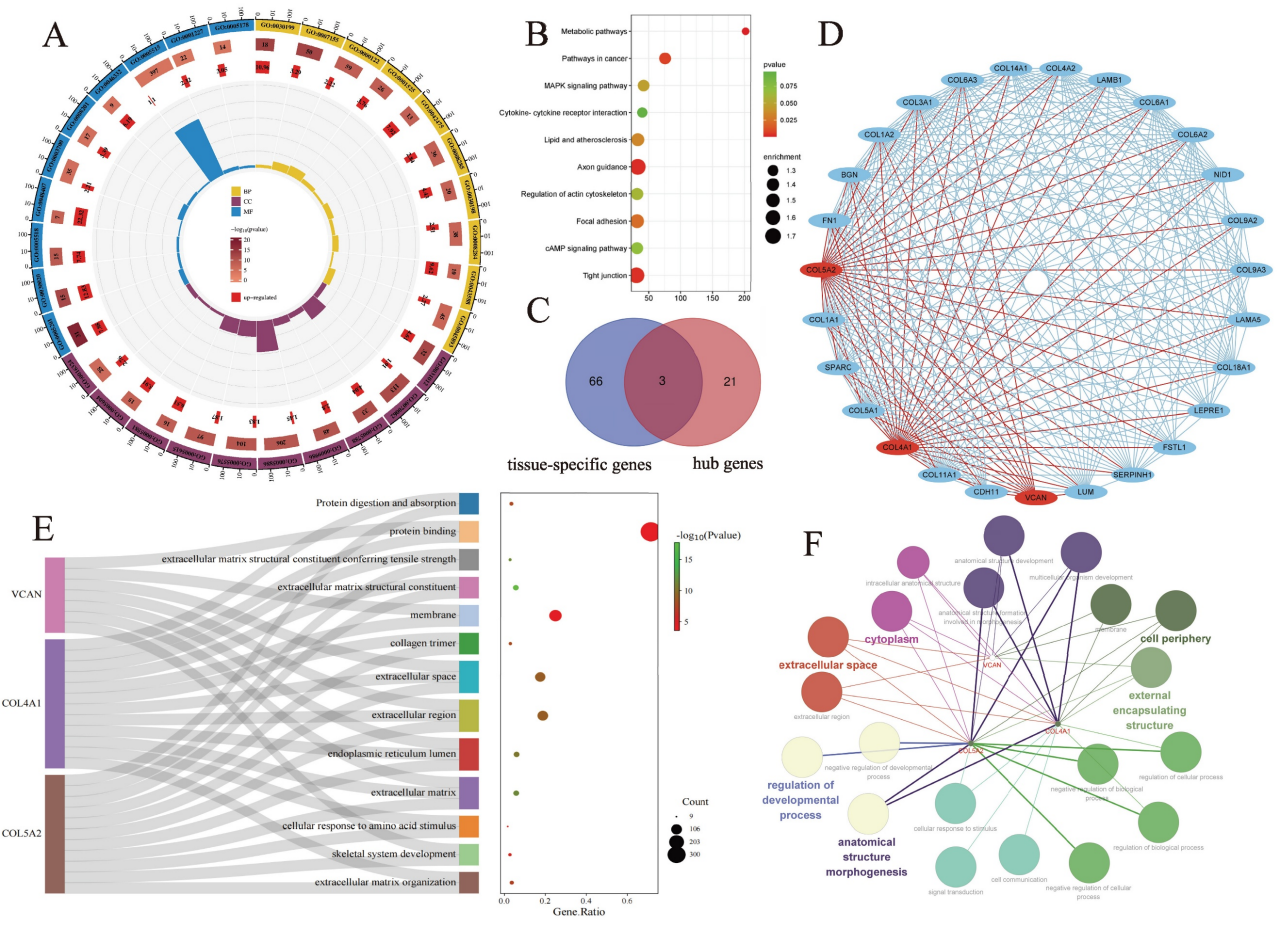
Enrichment analyses of EDGs. **A:** GO pathway enrichment analyses of 558 DEGs; **B:** KEGG pathway enrichment analyses of 558 DEGs; **C:** Venn diagrams between tissue-specific genes and hub genes; **D:** the most tightly clustered sub-networks were identified by the MCODE plugin, which was used to identify network gene clustering. The red node is the key tissue-specific genes we screened; **E:** Sankey diagram showing enrichment pathways related to key tissue-specific genes, the key tissue-specific genes participate in multiple key biological pathways; **F:** Interaction network laid out and visualized with ClueGO plugin of Cytoscape. Dots indicate the proteins interacting with the key tissue-specific genes, and the respective colours indicate the interaction types. The size of the node represents the number of interacting proteins, which belong to the respective gene ontology pathway.

Functional enrichment analysis of 558 DEGs was performed using the DAVID website. BP was mainly enriched in collagen fibril organization, cell adhesion, and angiogenesis. CC was mainly enriched in the extracellular matrix, extracellular exosome, endoplasmic reticulum lumen, cell surface, etc. MF mainly enriched in extracellular matrix structural component, extracellular matrix structural component conferring tensile strength and collagen binding. KEGG pathway enrichment analysis showed that DEGs were enriched in ECM-receptor interaction, Protein digestion and absorption, Focal adhesion, Basal cell carcinoma, Human papillomavirus infection (Fig. 2A, B).

The interaction network between the proteins encoded by the DEGs, consisting of 487 nodes and 1937 edges, was constructed using String and visualised using Cytoscape software. In this study, the most densely clustered subnetworks, consisting of 27 nodes and 280 edges, were identified using the MCODE plugin in Cytoscape. 24 hub genes were identified by intersecting the results of the five algorithms of Cytohubba, including MCC, DMNC, MNC, degree, EPC. We intersected 24 hub genes and tissue/organ specific expressed genes. Finally, three tissue-specific key genes were identified, including VCAN, COL4A1 and COL5A2 (Fig. 2C, D). Three key genes, VCAN, COL4A1 and COL5A2, may be involved in multiple activated signalling pathways in basal cell carcinoma, and they are co-enriched in extracellular space, extracellular region and endoplasmic reticulum lumen, extracellular matrix (Fig. 2E, F). Comparing our data with similar studies allowed us to minimize the presence of potentially confounding experimental design and technical differences. We compared our findings with the results already reported by Elizabeth RH *et al.* A significant increase in VCAN, COL4A1 and COL5A2 gene expression was also obtained in a gene expression profiling study of four BCC lesions and four corresponding matched control samples 32. Although the study was not initially included in the microarray analysis due to a sample size of less than 10, comparison with reported gene expression profiling helped to confirm the scientific validity and rigor of our study.

### Tumour-infiltrating immune cell analysis

**Figure 3:**
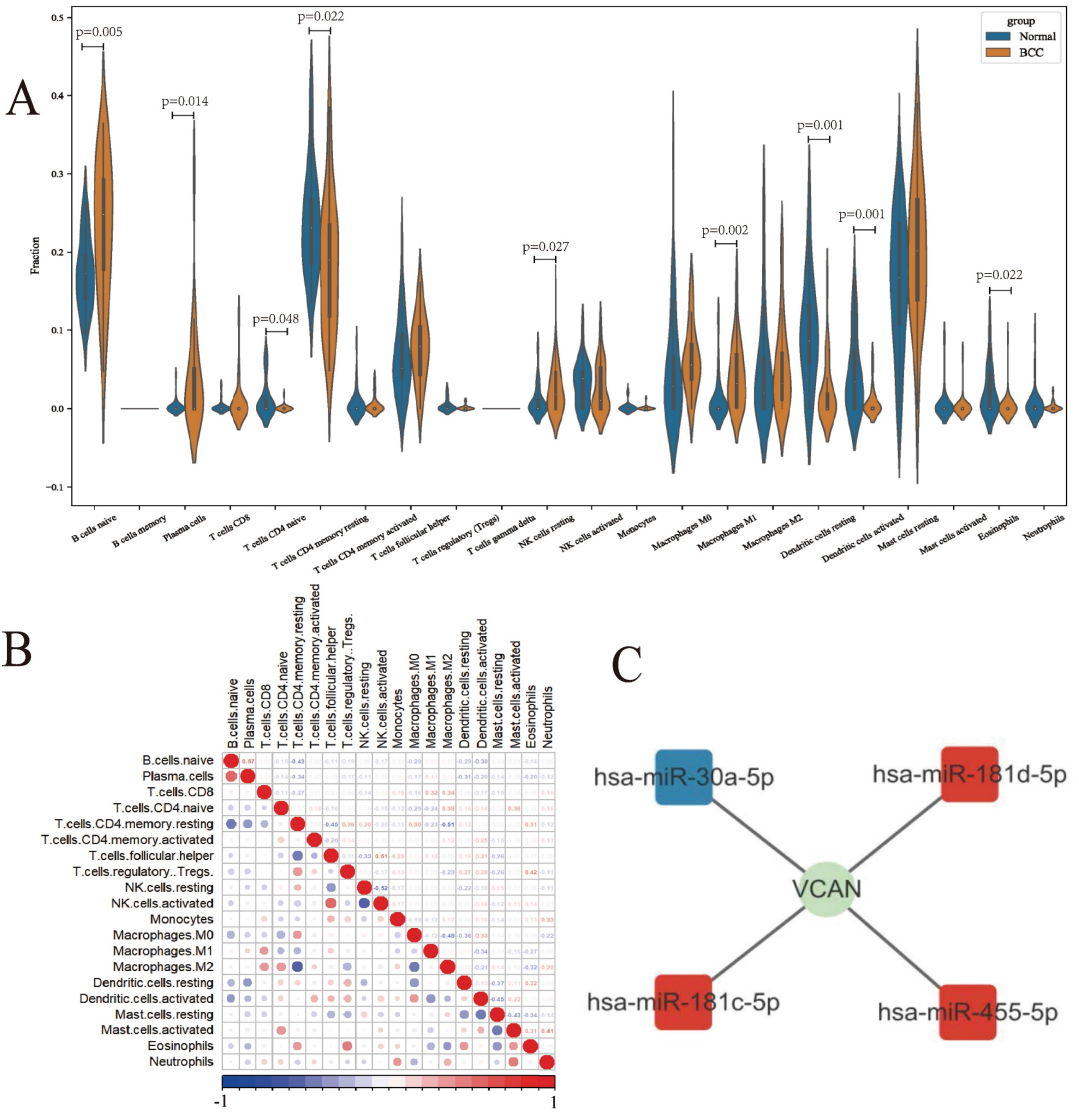
Composition of infiltrating immune cells assessed using the CIBERSORT algorithm in BCC tissues. **A:** Violin plot of infiltrating immune cells; **B:** Correlation among infiltrating immune cells; **C:** The immune-related hub ceRNA network. ns: not significant, *P < 0.05, **P < 0.01, ***P < 0.001.

Based on the GSE125285 dataset, immune cell distribution was assessed using the CIBERSORTx website. Using the validation dataset GSE53462, the results of infiltrating immune cells from GSE125285 were verified. (Fig. 3A, Fig. S3C) The analysis showed that resting NK cells (*P* = 0.027), M1 macrophages (*P* = 0.002) and activated dendritic cells (*P* = 0.001) were significantly up- and down-regulated, respectively, in BCC patient tissues compared to normal tissues. Immune cells with large positive correlation coefficients included naive B cells and plasma cells (0.57), follicular helper T cells and activated NK cells (0.51), regulatory T cells (Tregs) and eosinophils (0.42). Immune cells with large negative correlation coefficients included activated NK cells and resting NK cells (-0.52), M2 macrophages and resting CD4 memory T cells (-0.51), and M2 macrophages and M0 macrophages (-0.48) (Fig. 3B).

We predicted the possible upstream regulatory miRNAs of the VCAN gene and constructed a ceRNA network using miRNA datasets and prediction websites (Fig. 3C).

### Validation of the key tissue-specific genes

**Figure 4:**
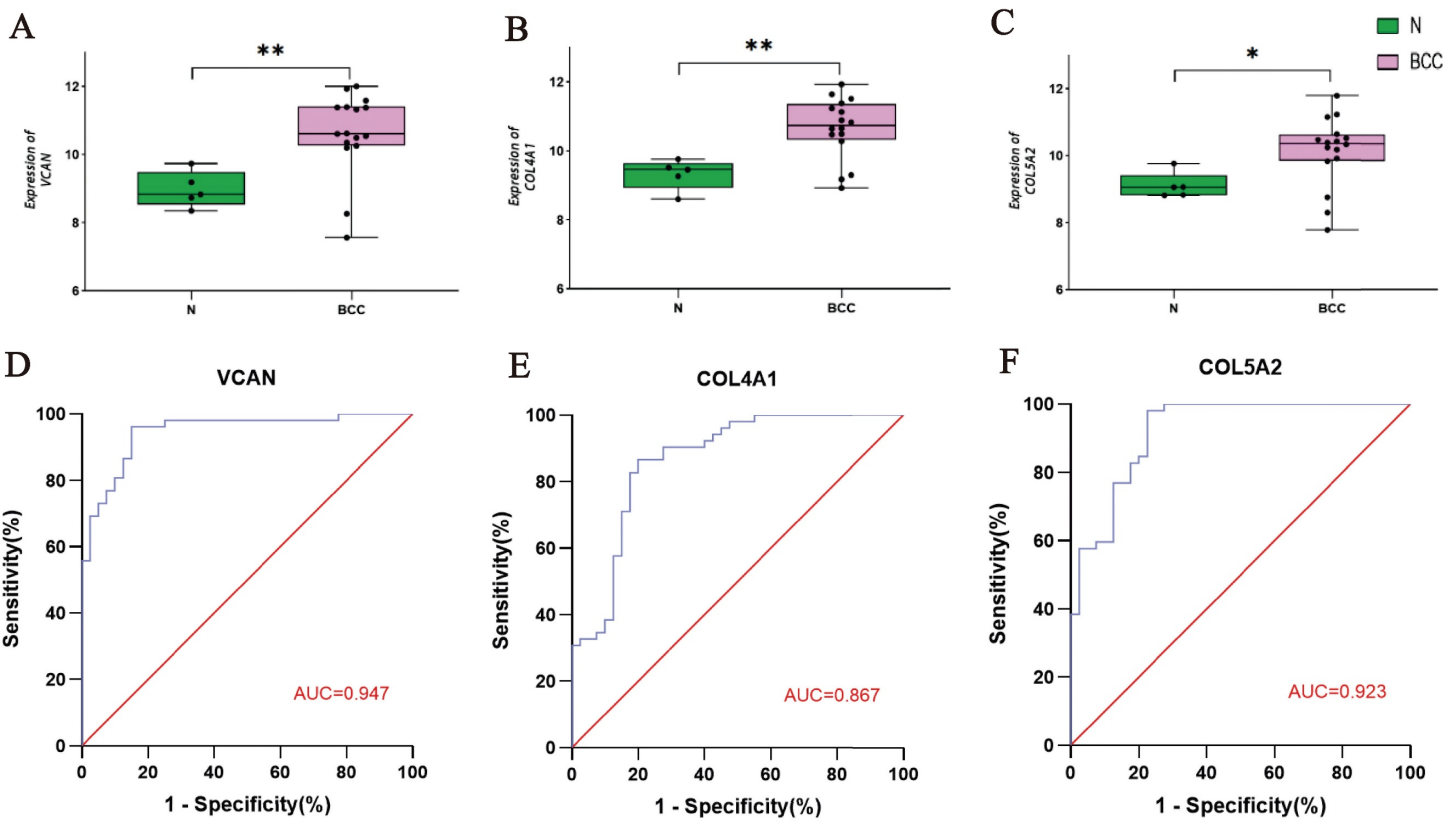
Validation of the Key Tissue-specific Genes and Construction of ROC Curves.** A-C:** Verifcation of the 3 key tissue-specific genes by the validation dataset GSE53462; **D-F:** ROC curve of the 3 key tissue-specific genes.

The GSE53462 dataset was used to validate the key tissue-specific genes and a box plot was generated. Consistent with our predictions, the mRNA expression levels of the three key tissue-specific genes were significantly increased in the BCC samples compared to the control samples (*P* < 0.05) (Fig. 4A-C). The results of the ROC curve reflect that these key tissue-specific genes have a high diagnostic value compared to control samples (Fig. 4D-F).

### VCAN promotes tumour cell proliferation *in vivo* and *in vitro*

**Figure 5:**
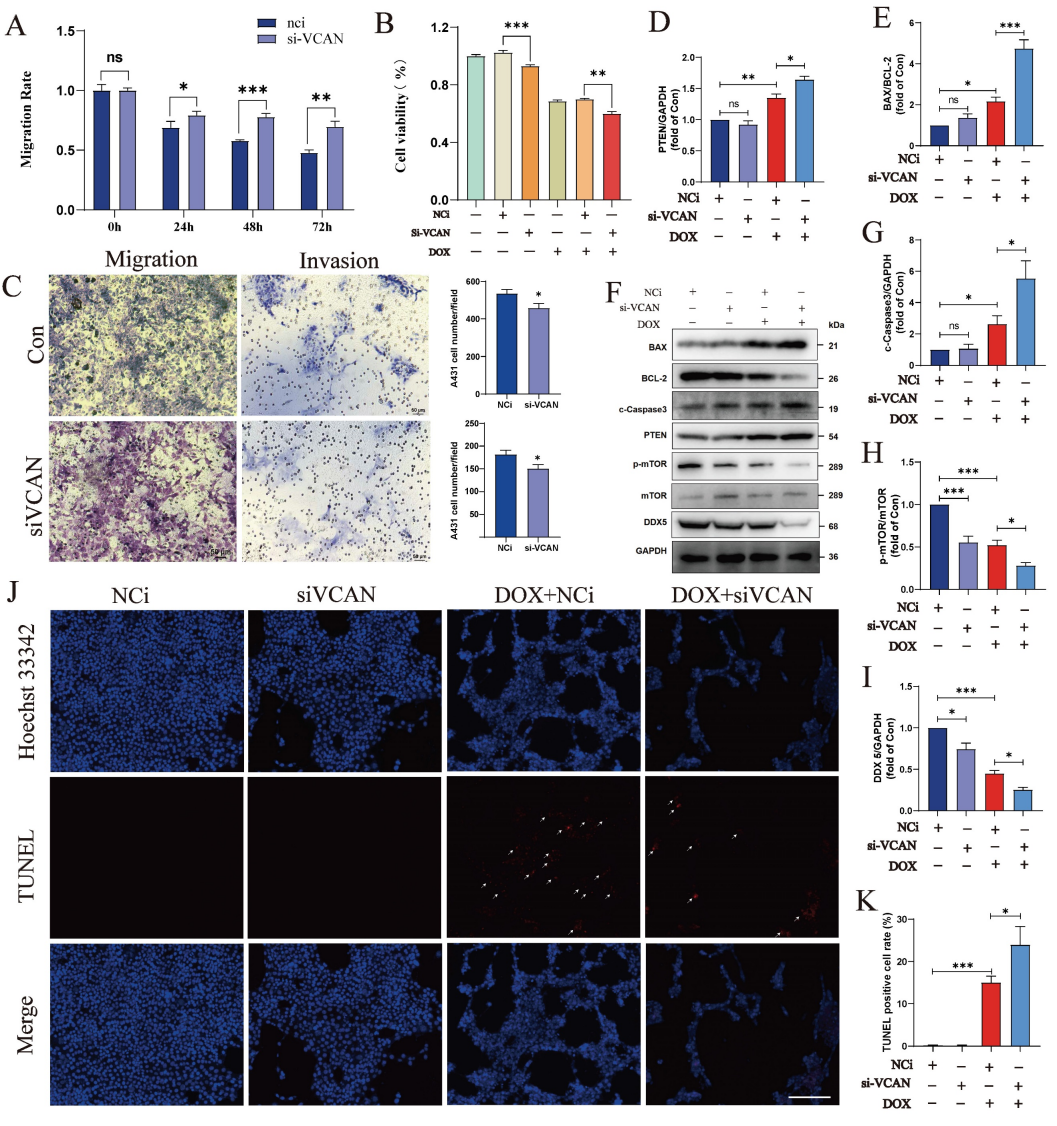
Silencing of VCAN regulates A431 cells proliferation, migration, invasion, and apoptosis. **A:** Cell migration rate was observed by wound healing assay at 24h, 48h and 72h; **B:** CCK-8 was used to detect the effect of VCAN on A431 cells proliferation under different treatments.** C:** 24h migration rate of A431(Top: Nci; Bottom: si-VCAN); **D-I:** Samples were collected from A431 cells after DOX treatment for 24h post-transfection, and the expression levels of total BAX, BCL-2, c-Caspase3, PTEN, phosphorylated and total mTOR, DDX5 and GAPDH were examined by Western blotting (n=3). **J, K**: Representative images of TUNEL and Hoechst 33342 staining of A431 cells (n=5). ns: not significant, *P < 0.05, **P < 0.01, ***P < 0.001.

To investigate the role of VCAN in tumours, we knocked down VCAN in A431 cells. Results from a wound-healing assay showed that suppression of VCAN in the A431 cell line resulted in a significantly lower rate of scratch closure than that observed in controls (Fig. 5A). CCK-8 assays showed that VCAN knockdown significantly inhibited the proliferation of A431 cell lines compared to the control group. Interestingly, after the addition of the anti-cancer drug DOX, VCAN knockdown can have a synergistic effect with DOX, which can further significantly inhibit the proliferation of A431 cells (Fig. 5B). Further studies showed that inhibition of VCAN with si-VCAN reduced the relative migration and invasion rates of tumour cells compared to the control group. These results suggest that inhibition of VCAN can inhibit BCC proliferation, invasion and migration *in vitro* (Fig. 5C). Western blotting analysis showed that VCAN knockdown enhanced DOX-induced apoptosis in A431 cells through the BAX/BCL-2/c-Caspase3 pathway.

**Figure 6:**
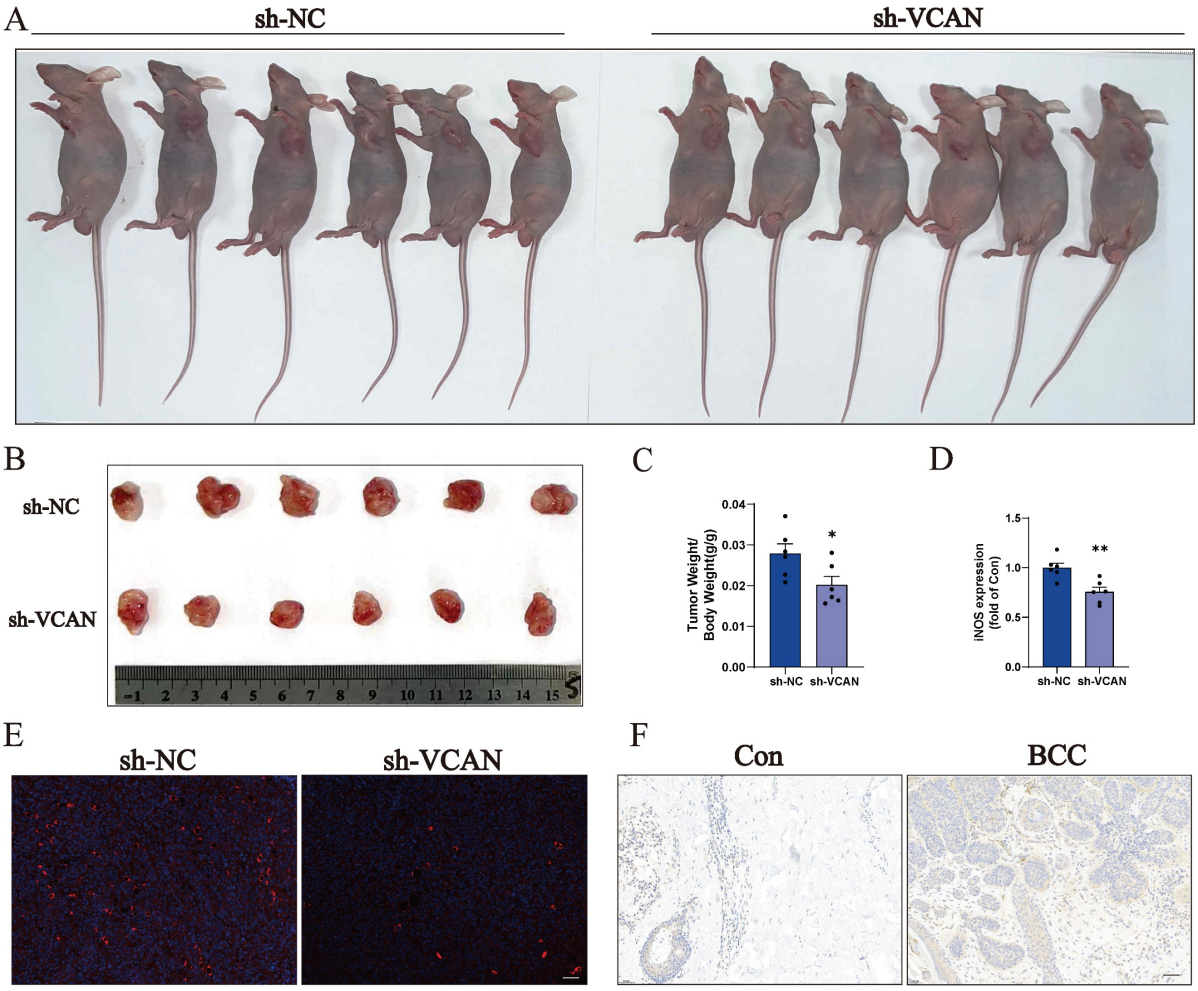
**A-C:** Nude mice were injected subcutaneously in the axillary region with 5×10^6^ control cells (sh-NC) or a VCAN knockout cell (sh-VCAN) for two weeks. Tumours were dissected and tumour/body weight calculated (n=6). Scale bar: 100 µm.** D, E**: Immunofluorescence of the macrophage M1 polarization marker iNOS (n=3). Scale bar: 100 µm. **F**: Immunohistochemical staining was used to analyse the expression levels of VCAN in normal human skin and tumour tissue from BCC patients (n=3). Scale bar: 50 µm. ns: not significant, *P < 0.05, **P < 0.01, ***P < 0.001.

Many transcription factors and transcription cofactors play important regulatory roles in basal cell carcinogenesis. Among them, DEAD-box RNA helicases play an important role in carcinogenesis and tumour metastasis, the most representative of which is DDX5. Accumulating evidence suggests that DDX5 plays a critical role in tumourigenesis, proliferation, metastasis, progression and drug resistance, which are potential biomarkers and targets 30, 33. We found that the expression of DDX5 was reduced when VCAN was knocked down; and the reduction of DDX5 was more significant when A431 treatment with DOX caused cell injury and death. Thus, VCAN may exert oncogenic effects by promoting DDX5 expression and inhibiting cell death through the BAX/BCL-2/c-Caspase3 pathway (Fig. 5D-I). TUNEL staining revealed that apoptosis was not significant when MCF-7 cells were treated with si-VCAN alone, but apoptosis was significantly increased in the co-treatment group (si-VCAN and 1 µM DOX) (Fig. 5J, K). In the *in vivo* experiments, cell derived xenograft model were performed in nude mice using control and VCAN knockdown tumour cells. Compared with the tumour cells in the control group, the tumour weight/body weight of the nude mice was significantly reduced 14 days after the VCAN knockdown tumour cells were injected subcutaneously into nude mice, and the results showed that the tumour size could be suppressed by VCAN knockdown, suggesting that VCAN is one of the key genes promoting tumour growth (Figure 6A-C). Previous studies have shown that increased expression of the VCAN gene facilitates the polarization of progenitor cells 34. Our analysis of immune infiltration in BCC patients also showed an increase in M1 macrophages. Therefore, we hypothesise that increased VCAN expression may promote M1 polarization of macrophages in BCC patients. Using animal samples for immunofluorescence staining analysis of M1 polarization markers, we observed a significant decrease in M1 polarization in the VCAN knockdown group compared to the control group (Figure 6D, E). Simultaneously, we validated VCAN expression in tumour samples from BCC patients and found that VCAN expression was higher than in normal skin samples, which was fully consistent with our bioinformatic findings (Figure 6F).

## Discussion

In this study, we identified 558 DEGs by comparing genes expressed in control tissues and BCC samples, including 69 tissue/organ-specific genes. According to GO enrichment analysis of the DEGs, extracellular matrix components were altered, resulting in increased cell adhesion and vascular proliferation. Notably, we found that KEGG annotation significantly enriched pathways associated with human papillomavirus infection. Human papillomavirus (HPV) is thought to be involved in the development of non-melanoma skin cancer (NMSC), especially when exposed to ultraviolet radiation, and HPV plays a synergistic role in the development of NMSC 35, 36. Francesca Paolini found the overexpression of p16INK4a and phospho-Akt, two proteins strongly associated with β-HPV 37. *In vitro* and *in vivo* studies of some cutaneous squamous cell carcinomas (cSCC) have shown that the E6 and E7 proteins of β-HPV can inhibit UV-induced cell cycle checkpoints and DNA repair, inactivate p53 and cause cell immortalisation 38. However, there is a lack of research on BCC. Some studies have also found that there is no correlation between HPV and the occurrence of BCC. Iannacone's research showed that HPV is not involved in the carcinogenesis of BCC. HPV may play an important role in the carcinogenesis of squamous keratinocytes, but not in basal cell keratinocytes 39. Therefore, the role of HPV in the pathogenesis of BCC and the regulatory mechanisms need to be further investigated.

In the GSEA analysis, we found that the gene sets associated with the Hedgehog pathway were significantly enriched. The Hedgehog pathway has been demonstrated to be involved in the progression and worsening of BCC. Hedgehog pathway inhibitors, most notably vismodegib and sonidegib, which have been approved by the US Food and Drug Administration (FDA) and the European Medicines Agency (EMA), have become the standard systemic treatment for BCC patients with locally advanced lesions, metastases or unresectable cancer. GSEA of 25 samples from BCC patients and 25 samples from healthy controls also showed that UV radiation, as an inducer of BCC, promotes keratin formation in the skin.

In BCC, necrotic tissue and abnormally proliferating tumour tissue activate immune cells and act as foreign bodies to trigger an immune response. We analysed 22 immune cells from 50 samples using the CIBERSORT algorithm and identified alterations in the immune microenvironment of BCC patients. Resting NK cells, M1 macrophages were significantly up-regulated and activated dendritic cells were significantly down regulated in the tissues from BCC patients compared to normal tissues. Macrophages are mainly divided into M1 and M2 subpopulations. M1 macrophages have a strong antigen-presenting capacity and generate a strong immune response against cancer cells, exerting an anti-tumour effect. M2 macrophages are mainly involved in clearing the immune response, inducing angiogenesis and tissue repair, and exerting pro-tumour activity 40. We found that M1 macrophages were significantly increased in BCC patient samples, indicating a strong anti-tumour immune response. Similar to our findings, Nestle FO *et al.* found that dendritic cells were not activated due to lack of stimulation of effector T cells and their antigen presentation function was impaired in artificially induced BCC 41. Therefore, we considered that M1 macrophages may have a crucial anticancer role in BCC patients, but the tumour-immune mechanism requires in-depth study. Previous studies have shown that VCAN may be associated with M1 macrophage polarization in the tumour microenvironment 11. To verify the relationship between VCAN and M1 macrophage polarization, we performed fluorescence staining of tumour sections from cell-derived xenograft mouse models. M1 macrophage polarization was significantly reduced in tumour tissue after VCAN knockdown compared to control tumours, consistent with bioinformatic predictions. This suggests that VCAN can indeed affect the polarization of tumour-associated macrophages.

Through gene enrichment analysis, we found that three key tissue/organ-specific genes are involved in a variety of physiological and pathological processes. Collagen is the most abundant protein in the extracellular matrix and an important component of the tumour microenvironment. Aberrant collagen expression affects the behaviour of tumour cells. COL4A1 encodes type IV collagen alpha protein, which is involved in the formation of the extracellular matrix and whose mutation can lead to cerebrovascular disease or muscle defects. COL4A1 is the most over-expressed collagen gene in HCC and promotes HCC proliferation, migration and invasion through the FAK-Src signalling pathway 42. COL5A2 encodes type V collagen alpha protein, which is associated with type XI collagen. The V and XI collagen chains can form a single collagen type with tissue-specific chain combinations. COL5A2 has been associated with prognosis in tongue cSCC, oesophageal cSCC, bladder cancer and ovarian cancer 43. Thus, COL4A1 and COL5A2 are both important components of the extracellular matrix and may have great clinical potential in BCC.

VCAN, a type of aggregated chondroitin sulphate proteoglycan, is a member of the aggregation proteoglycan family. This protein plays a key role in histomorphogenesis and maintenance, and is involved in cell adhesion, proliferation, migration, and angiogenesis. VCAN interacts with a variety of extracellular matrix (ECM) components, including tumour necrosis factor-stimulated gene 6 (TSG-6), fibronectin, tendon protein and CD44. One of the most studied interactions is that between the amino-terminal structural domain of VCAN and hyaluronic acid. There are at least four sources of VCAN production in tumour tissue: tumour cells, stromal cells, tumour-associated bone marrow cells, and possibly tumour-infiltrating lymphocytes 44. VCAN is an epithelial-mesenchymal transition-associated gene that promotes the development of hepatocellular carcinoma (HCC), breast cancer, non-small cell lung cancer, etc 45. Karvinen S noted that some BCC samples were found to be positive for VCAN staining at peritumoural mesenchymal sites 46. Previous studies have shown that VCAN stimulates tumour cell proliferation through two main mechanisms: 1. promotion of cell growth through two EGF-like sequences in the G3 structural domain; 2. disruption of cell adhesion and promotion of cell growth through the G1 structural domain 44. Tumours overexpressing the G3 structural domain not only had high levels of 4B6, pEGFR, pAKT, and GSK-3β (S9P), all of which are associated with tumour aggressiveness, but also exhibited high levels of the tumour stem cell markers Sox2, Sca-1, and ALDH1 47. Some study also found that VCAN is associated with metastasis in a variety of cancers 48. In our study, we found that VCAN knockdown reduced the proliferation, migration and invasion ability of BCC and the cell-derived xenograft model study showed that knockdown of VCAN in tumour cells significantly suppressed tumour size. Meanwhile, the expression of VCAN in tumour samples from BCC patients was significantly higher than that in normal skin tissues. These results confirm the potential use of VCAN in the diagnosis and treatment of BCC.

Recent studies have revealed a close and complex relationship between VCAN expression and tumour cell apoptosis. It has been shown that VCAN can exert anti-apoptotic effects by increasing cell adhesion, cell-matrix interactions and the expression of integrin β1 and fibronectin, thereby protecting cells from oxidative stress-induced apoptosis in tumour cells. This is consistent with our finding that VCAN knockdown promotes apoptosis and reduces drug resistance in skin cancer cells. However, VCAN has also been shown to have pro-apoptotic effects in certain tumours. One study showed that knockdown of VCAN blocked the promotion of apoptosis by the G3 structural domain in a human breast cancer cell line 47. The inconsistent and controversial results of existing studies on the role of VCAN in regulating apoptosis in tumours suggest that the complexity of VCAN in regulating apoptosis during tumourigenesis and progression may be due to differences in regulatory pathways in different tumours, and further studies are needed to verify this.

In this study, we identified VCAN as a key gene affecting the proliferate, migrate, and invade ability of BCC *in vivo* and *in vitro*. However, there are still some limitations. Primarily, Keiichi Asano *et al.* found that VCAN preferentially localized in the proximity of tumour blood vessels and macrophages in tumours 5. VCAN haploinsufficiency impaired proper tumour vessel invasion, which suggests that VCAN contributes to tumour angiogenesis in stromal tissues, and the role of VCAN in angiogenesis in BCC is also a direction we need to investigate in the future.

## Conclusions

We have screened and identified the key tissue/organ specific gene VCAN and found that the expression of VCAN is positively correlated with the occurrence of BCC and promotes the proliferation and migration of tumour cells. The cell-derived xenograft model study in nude mice showed that knockdown of VCAN in tumour cells significantly suppressed tumour size. VCAN expression was found to be significantly higher in BCC patient samples than in normal human skin samples. These results suggest that VCAN may be used as a potential clinical target for the diagnosis and treatment of BCC.

## Supplementary Material

Supplementary figures.

## Figures and Tables

**Table 1:** Information for selected microarray datasets

	Datasets	Participants	Platform	Attribute
miRNA	GSE34535	7 BCC and 7 controls	GPL15019	Training set
	GSE34137	6 BCC and 6 controls	GPL10999	Training set
mRNA	GSE125285	25 BCC and 25 controls	GPL11154	Training set
	GSE42109	11 BCC and 10 controls	GPL570/GPL571	Training set
	GSE53462	16 BCC and 5 controls	GPL10558	Validation set

Annotation: GPL15019: Agilent-031181 Unrestricted Human miRNA V16.0 Microarray 030840 (miRBase release 14.0 miRNA ID version); GPL10999: Illumina Genome Analyzer IIx (Homo sapiens); GPL11154: Illumina HiSeq 2000 (Homo sapiens); GPL570: [HG-U133 Plus 2] Affymetrix Human Genome U133 Plus 2.0 Array; GPL571: [HG-U133A 2] Affymetrix Human Genome U133A 2.0 Array; GPL10558: Illumina HumanHT-12 V4.0 expression beadchip.

**Table 2:** Distribution of tissue/organ-specific expressed genes identified by BioGPS

System/Organ	Genes	Counts
Haematologic/Immune		
Haematologic/Immune cells	CCND2, SPON2, RORA, LGALS8, CYP1B1, VCAN, KLF4, SORL1, AQP9, SOX4, PRAME, NMU, SOCS1	15
Immune organs	LEF1, CD1A	
Nervous	TPD52L1, SHROOM2, CDHR1, PLCH2, PRKAR1B, COBL, FBXW7, SOX11, TRO, NAV2, NREP, LHX2, MLLT11, COCH	14
Respiratory	MAP3K6, CLDN5, ZBTB16, FMO2, SCNN1, BCDH3, GJB3, ANXA3, SFN	9
Placenta	MMP11, TUSC3, TPPP3, EPAS1, COL4A1, EXPH5, EXPH5	7
Endocrine	C2CD2, SLC47A1, CA6, GPC4, BSPRY, NBL1	6
Muscle	SLC39A14, MFAP2, COL5A2, SYNC, PXDN, PCNT	6
Digestive	CHP2, OLFM4, PTK6, CHGA, HMGCS2	5
Colorectal adenocarcinoma	ANO1, MST1R, KRT23	3
Tongue	LY6D, EVPL	2
kidney	CA12	1
Circulatory	SGCG	1

## Abbreviations

BCC: Basal cell carcinoma
NMSC: non-melanoma skin cancer
GEO: Gene Expression Omnibus
DEG: Differential Expressed Gene
AUG: area under the curve
ncRNA: noncoding RNA
lncRNA +: long non-coding RNA
circRNA: circular RNA
miRNA: microRNA
FDR: False Discovery Rate
GO: Gene Ontology
KEGG: Kyoto Encyclopedia of Genes and Genomes
ceRNA: Competitive endogenous RNA
PPI: protein-protein interaction
BP: biological processes
CC: cellular components
MF: molecular functions
GSEA: Gene Set Enrichment Analysis
MCODE: Molecular Complex Detection
MCC: Maximal Clique Centrality
MNC: Maximum Neighborhood Component
DMNC: Density of Maximum Neighborhood Component
EPC: Edge Percolated Component
DCs: dendritic cells
NKs: natural killer cells
Tregs: regulatory T cells
Tfhs: T follicular helper cells
Tgds: gamma delta T cells
cSCC: cutaneous squamous cell carcinoma
FDA: the US Food and Drug Administration
EMA: the European Medicines Agency
HCC: hepatocellular carcinoma
HMG: high mobility group
NSCLC: non-small cell lung cancer
SSI: the STAT-induced STAT inhibitor
