# Supplementary material for: Screening and identification of versican as a sensitive biomarker and potential therapeutic target in basal cell carcinoma
Source: Int J Med Sci. 2025 Apr 28;22(10):2488–501. doi: 10.7150/ijms.105650 (PMC12080574; doi:10.7150/ijms.105650)

Supplementary materials:

Screening and identification of versican as a sensitive biomarker and potential therapeutic target in basal cell carcinoma

Wenlin Li[1, 2#], Yang Wang[3#], Qiang Hu[1, 4], Sainan Li[2, 4], Dachuan Guo[2, 4], Lin Liu[1, 4], Xiuqing Huang[2], Lin Dou[2], Qi Zhou[2*], Tao Shen[2*], Jianmin Chang[1, 4*]

[1] Department of Dermatology, Beijing Hospital, National Center of Gerontology, Institute of Geriatric Medicine, Chinese Academy of Medical Sciences, Beijing, People's Republic of China

[2]The Key Laboratory of Geriatrics, Beijing Institute of Geriatrics, Institute of Geriatric Medicine, Chinese Academy of Medical Sciences, Beijing Hospital/National Center of Gerontology of National Health Commission, Beijing, China, 100730

[3]School of life sciences, North China University of Science and Technology, Tangshan 063210, China

[4]Peking Union Medical College, Chinese Academy of Medical Sciences, Graduate School of Peking Union Medical College, Beijing, People's Republic of China

[#] These authors contributed equally to this work

*To whom correspondence should be addressed: Jianmin Chang, changjianmin@medmail.com.cn, Tel: +86-010-85133303; Qi Zhou, Email: zhouqi4738@bjhmoh.cn; Tao Shen, Email: shentao4189@bjhmoh.cn.

Supplementary Figure 1. Heat maps and volcano plots of DEGs. **A:** GSE34535; **B:** GSE34137; **C:** GSE125285; **D:** GSE42109.

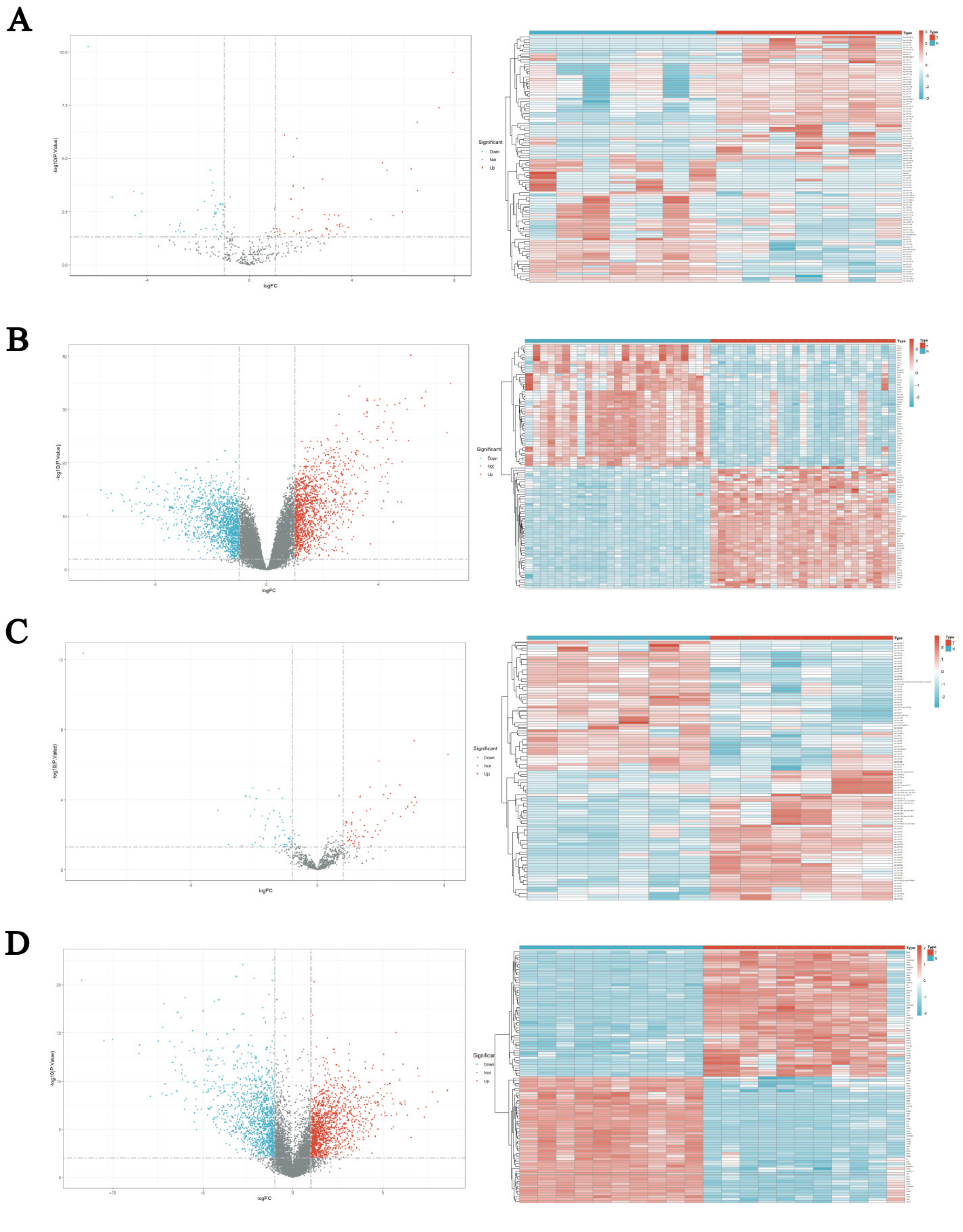

Supplementary Figure 2. GSEA plot showing the most enriched BCC-related genes sets in the dataset GSE125285 and GSE42109. From left to right**:** Gene set enrichment analysis: PID HEDGEHOG 2PATHWA; Gene set enrichment analysis: MURAKAMI UV RESPONSE 24HR; Gene set enrichment analysis: REACTOME GEHOG ON STATE; Gene set enrichment analysis: KEGG HEDGEHOG SIGNALING PATHWAY; Gene set enrichment analysis: HP BASAL CELL CARCINOMA (above: GSE125285, below: GSE42109). The screening criteria for significant gene sets were *P* < 0.05 and Q < 0.25. NES: normalized enrichment score; *P*-vale: also named False Discovery Rates (FDR)or adjust *P* value.

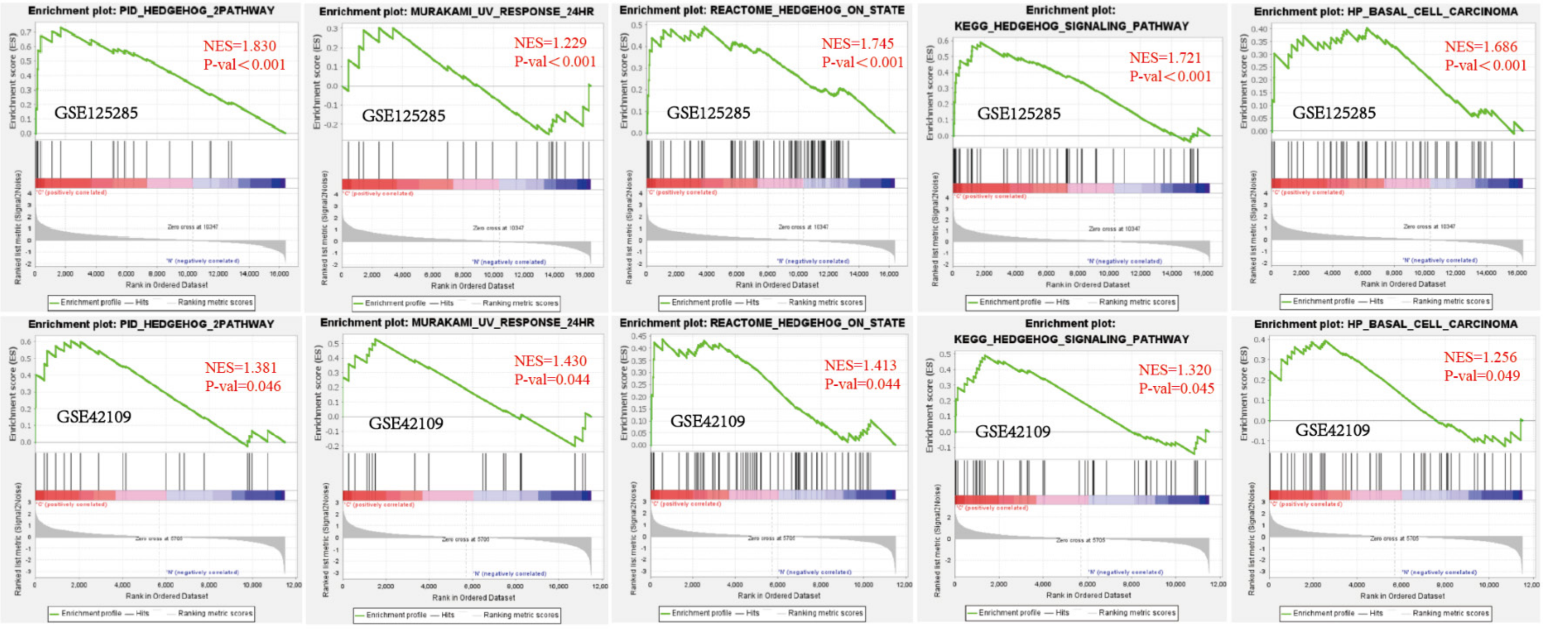

Supplementary Figure 3. Composition of infiltrating immune cells assessed using the CIBERSORT algorithm in BCC tissues by the validation dataset GSE53462. **A:** Distribution of immune cell infiltration in each sample; **B:** Heatmap of immune cell types; **C:** Violin plot of infiltrating immune cells.

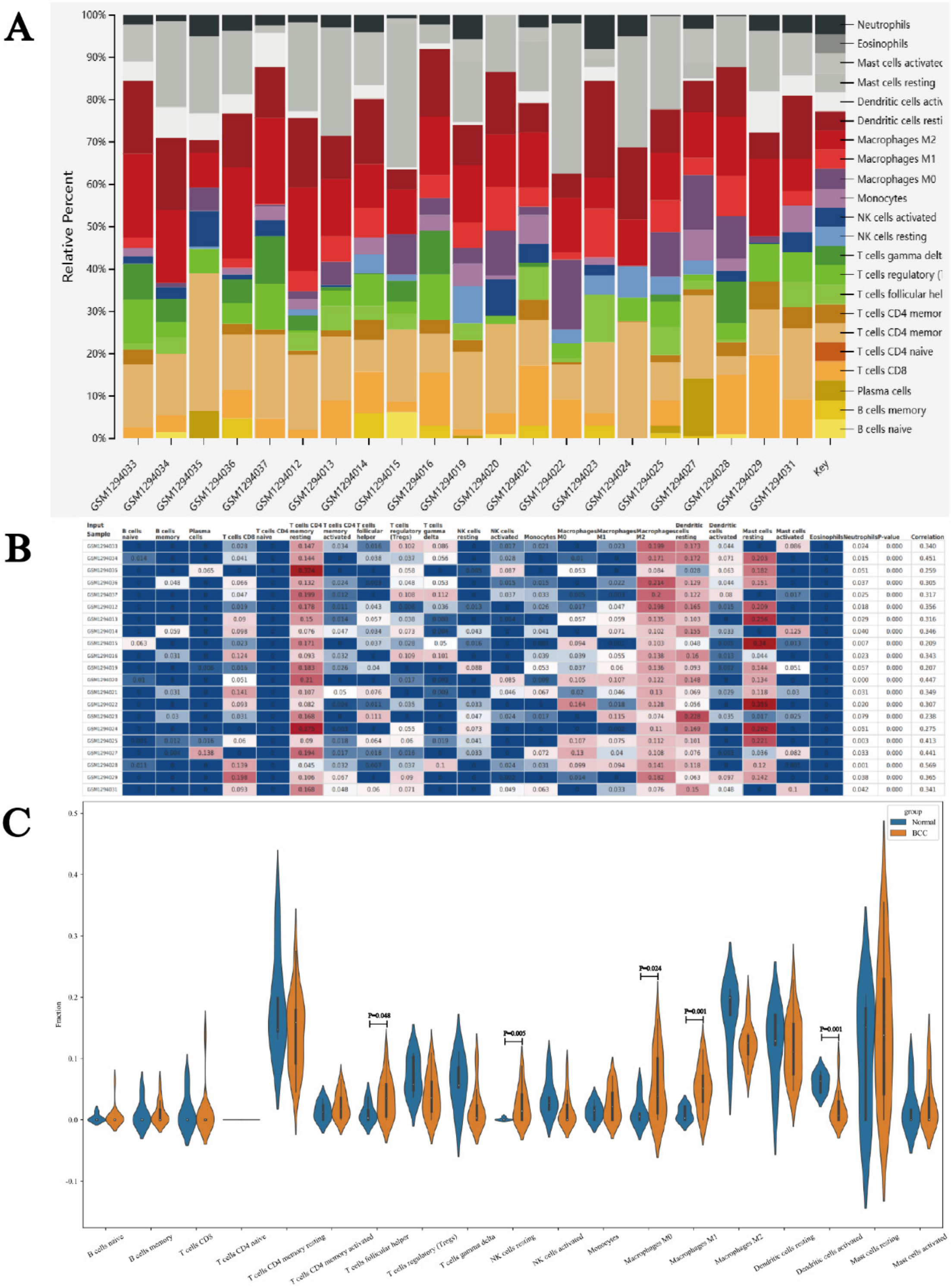
A
Relative Percent
100%
90%
80%
70%
60%
50%
40%
30%
20%
10%
0%
GSM1294033
GSM1294034
GSM1294035
GSM1294036
GSM1294037
GSM1294012
GSM1294013
GSM1294014
GSM1294015
GSM1294016
GSM1294019
GSM1294020
GSM1294021
GSM1294022
GSM1294023
GSM1294024
GSM1294025
GSM1294027
GSM1294028
GSM1294029
GSM1294031
Key
Neutrophils
Eosinophils
Mast cells activatec
Mast cells resting
Dendritic cells activ
Dendritic cells resti
Macrophages M2
Macrophages M1
Macrophages M0
Monocytes
NK cells activated
NK cells resting
T cells gamma delt
T cells regulatory (
T cells follicular hel
T cells CD4 memor
T cells CD4 memor
T cells CD4 naive
T cells CD8
Plasma cells
B cells memory
B cells naive
B
Input Sample
B cells naive
B cells memory
Plasma cells
T cells CD8
T cells CD4 naive
T cells CD4 memory resting
T cells CD4 memory activated
T cells follicular helper
T cells regulatory (Tregs)
T cells gamma delta
NK cells resting
NK cells activated
Monocytes
Macrophages M0
Macrophages M1
Macrophages M2
Dendritic cells resting
Dendritic cells activated
Mast cells resting
Mast cells activated
Eosinophils
Neutrophils
P-value
Correlation
C
Fraction
group
Normal
BCC
P=0.048
P=0.005
P=0.024
P=0.001
P=0.001
B cells naive
B cells memory
T cells CD8
T cells CD4 naive
T cells CD4 memory resting
T cells CD4 memory activated
T cells follicular helper
T cells regulatory (Tregs)
T cells gamma delta
NK cells resting
NK cells activated
Monocytes
Macrophages M0
Macrophages M1
Macrophages M2
Dendritic cells resting
Dendritic cells activated
Mast cells resting
Mast cells activated

Supplementary Figure 4. Tissue-specific pattern of mRNA expression of key genes from the online tool BioGPS.

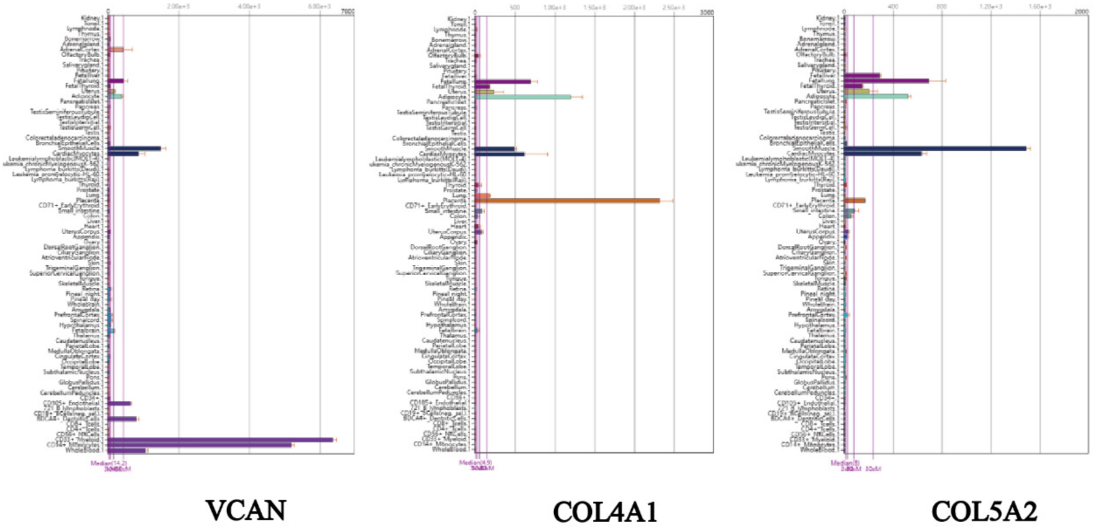

Supplement: Supplementary file 1 — Supplementary figures. [file ijmsv22p2488s1.pdf]
